# Enhanced response and sensitivity of self-corrugated graphene sensors with anisotropic charge distribution

**DOI:** 10.1038/srep11216

**Published:** 2015-06-08

**Authors:** Seung Yol Jeong, Sooyeon Jeong, Sang Won Lee, Sung Tae Kim, Daeho Kim, Hee Jin Jeong, Joong Tark Han, Kang-Jun Baeg, Sunhye Yang, Mun Seok Jeong, Geon-Woong Lee

**Affiliations:** 1 Nano Hybrid Technology Research Center, Korea Electrotechnology Research Institute (KERI), Changwon 642-120, Republic of Korea; 2 Multidimensional Nanomaterials Research Group, Korea Electrotechnology Research Institute (KERI), Changwon 642-120, Republic of Korea; 3 Perform Modeling Research Division, Korea Institute of Carbon Convergence Technology, Jeonju 561-844, Republic of Korea; 4 Center for Nanostructure Physics (CINAP), Institute for Basic Science (IBS), WCU Department for Energy Science, Sungkyunkwan University, Suwon 440-746, Republic of Korea; 5 Department of Electrical Functionality Material Engineering, University of Science and Technology (UST), Daejon 305-333, Republic of Korea; 6 Department of Chemistry & Chemical Engineering, Inha University, Incheon 402-751, Republic of Korea

## Abstract

We introduce a high-performance molecular sensor using self-corrugated chemically modified graphene as a three dimensional (3D) structure that indicates anisotropic charge distribution. This is capable of room-temperature operation, and, in particular, exhibiting high sensitivity and reversible fast response with equilibrium region. The morphology consists of periodic, “cratered” arrays that can be formed by condensation and evaporation of graphene oxide (GO) solution on interdigitated electrodes. Subsequent hydrazine reduction, the corrugated edge area of the graphene layers have a high electric potential compared with flat graphene films. This local accumulation of electrons interacts with a large number of gas molecules. The sensitivity of 3D-graphene sensors significantly increases in the atmosphere of NO_2_ gas. The intriguing structures have several advantages for straightforward fabrication on patterned substrates, high-performance graphene sensors without post-annealing process.

Molecular detection using graphene is a promising method for use in gas sensors and biosensors because of its outstanding electrical properties and large specific surface area^1^,^2^. This approach is also applicable in compact, low-power, and flexible portable sensors. In order to produce high-performance molecular sensors, several characteristics must be balanced, including sensitivity, selectivity, reversible fast response with saturation region, fast recovery time, and room-temperature operation. In general, resistive-type molecular sensing is governed by charge transfer between sp^2^ carbon and gas molecules. Specifically, graphene-based gas sensors can detect individual molecular levels of gas adsorption^3^,^4^,^5^. This mechanism operates via charge transfer, which is induced by resistance changes during adsorption and desorption of molecules. Here, the gaseous molecules act as an electron donor or acceptor on graphene surfaces. Thus, the large surface area and high electrical conductivity of graphene materials can increase the charge transfer because of their abundant active sites and fast carrier transport^3^,^6^. To date, graphene, as a two-dimensional material, has been prepared by various methods such as mechanical exfoliation, epitaxial growth, chemical vapor deposition (CVD), and chemical exfoliation^7^,^8^,^9^,^10^. As a practical approach, CVD and chemical exfoliation have attracted considerable attention because of their simple nature and large-scale production capabilities. In particular, reduced graphene oxide (rGO), resulting from the chemical exfoliation and reduction of graphite, is a possible candidate for flexible and wearable electronics because of its solution-based processing and low cost^11^,^12^. In turn, rGO can be easily and efficiently applied to continuous-process applications such as screen printing, ink-jet printing, gravure, roll-to-roll, spray methods, etc. However, rGO is rendered with various stoichiometries and local arrangements of chemical functional groups, which can alter the intrinsic properties of graphene^13^. Because of these drawbacks, rGO exhibits poor performance in terms of molecular detection. Thus, it has been suggested that an evaluation be performed on the sensing properties of rGO in terms of annealing at high temperature as a drastic reduction using macro-, micro-, and nano-structured rGO—for instance, three-dimensional (3D) graphene foam, etched porous graphene oxide (GO), graphene ribbons, graphene–metal oxide hybrid materials, and carbon nanotubes—for the formation of large surface areas with high electrical conductivity^14^,^15^,^16^,^17^,^18^. Despite outstanding progress with rGO-based materials, these approaches are still lacking for the practical use in terms of high-performance molecular sensing, reversible fast response to adsorption and desorption of gaseous molecules, large-scale fabrication by simple methods, and room-temperature operation. In addition, high-temperature annealing or UV irradiation is required to render an effective operation of devices. This is due to the limiting factors of electron mobility during unstable charge transfer on graphene layers, in terms of mechanical or chemical defects and charge impurities.

Thus, the goal here is to develop an easily fabricated, high-performance rGO molecular sensor capable of room-temperature operation, and, in particular, exhibiting high sensitivity and reversible fast response with saturation region. For this purpose, we introduce self-corrugated, chemically modified graphene as a 3D structure with anisotropic charge distribution. The accumulated electrons on the corrugated-edge area of the graphene layers exhibit a high electric potential compared with two-dimensional rGO (2DrGO) films. This local accumulation of electrons interacts with a large number of gas molecules, resulting in a reversible faster response and higher sensitivity than with conventional rGO sensors. The density of states of the self-corrugated graphene surface was confirmed by Scanning Kelvin Force Microscopy (SKFM), and the gradient of electric potential as a function of wall height on the 3DrGO was modeled by computational simulation as a function of anisotropic charge distribution. The field-enhancement factor is five times higher than that of 2D graphene film, and a fast response with saturation of resistance change was confirmed for the adsorption and desorption of NO_2_ gas molecules. Moreover, the sensitivity of the gas sensor increased significantly at various NO_2_ gas concentrations.

## Results and discussion

**Figure 1:**
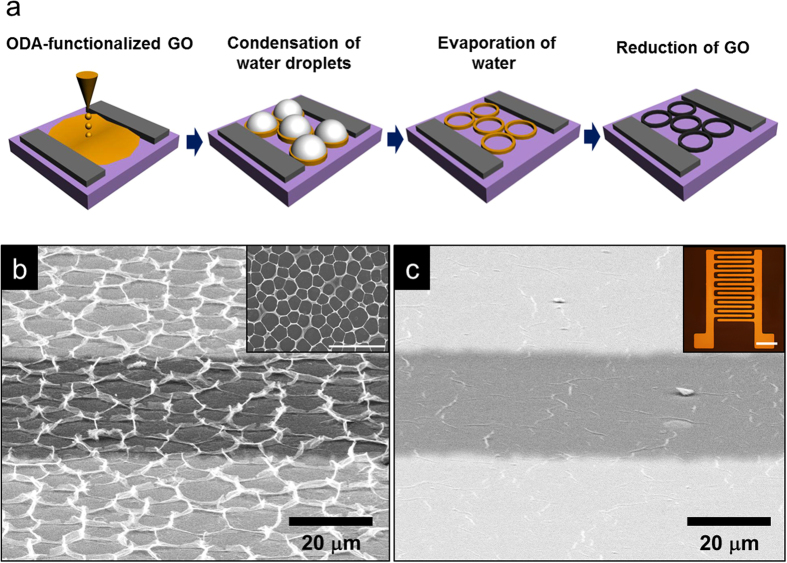
(**a**) Schematic diagram of the preparation of 3DrGO on patterned electrodes by the “breath-figure” method. (**b**) FE-SEM image of self-corrugated 3DrGO with a 45° tilted view between two metal electrodes (scale: 20 μm); inset: top view of the 3DrGO (scale: 100 μm). (**c**) Tilted FE-SEM image of 2DrGO without condensation and evaporation of water; inset: interdigitated metal electrode on a SiO_2_ substrate with a 300 nm oxide-layer thickness (scale bar: 500 μm).

The Fig. 1a illustrates the procedure for producing self-corrugated graphene on patterned electrodes by the breath-figure method^19^. An octadecylamine-(ODA) functionalized GO solution in toluene with a concentration of 1.5 g/L (added drop-wise) was spin-cast on a patterned electrode with a SiO_2_ thickness of 300 nm for the fabrication of a resistive-type gas sensor. In order to produce a well-aligned, corrugated rGO structure, the GO film was placed in a prepared chamber with a relative humidity of 80%. The rapid evaporation of toluene reduces the temperature at the air–solution interface because the toluene is highly volatile and immiscible with water vapor. Thus, condensation and nucleation of micro-sized water droplets results on the substrate. Rapid evaporation and airflow across the surface of the ODA-functionalized GO solution led to convective flows in the solution; the water droplets then created regular corrugation by packing of GO nanosheets. The GO nanosheets encapsulate the water droplets and precipitate at the water–solution interface. Following the evaporation of water from the GO nanosheets, self-corrugated regular vertical GO structures were produced on their surfaces. It has been established that solvents with a low viscosity have a low surface energy and their droplets have a tendency to adopt a spherical shape on the surface of high-viscosity solvents to minimize surface energy^20^. In general, the surface tension of water and toluene at room temperature are 71.97 and 27.73 mN/m, respectively^21^. Though the viscosity of water is higher than that of toluene, the viscosity of the ODA-functionalized GO in toluene is higher than that of water, owing to the addition of solutes. Thus, the condensation of water droplets occurs on the GO surfaces. The prepared GO structure then underwent GO reduction via fuming hydrazine (N_2_H_4_) at 80 °C overnight. The both materials are deposited on the same interdigitated structure. This process constitutes a simple means for the fabrication of resistive-type rGO-based devices with various flexible substrates. Figure 1b shows the morphology of self-corrugated graphene on the patterned substrates as a 3DrGO structure. Conventional 2DrGO nanosheets were formed on the patterned electrodes shown in the inset of Fig. 1c. The interdigitated pattern shown in this inset was fabricated by thermal evaporation of Cr and Au, with thicknesses of 20 nm and 100 nm, respectively, on SiO_2_ at thickness of 300 nm. The length and width of interdigitated electrodes are 50 mm, respectively. Figure 1c shows rGO nanosheets on the patterned substrates without self-corrugation, which represents a 2DrGO structure. In order to intensively compare the effects of structural changes to rGO, 2DrGO was also prepared by ODA-functionalized GO without further evaporation in humid conditions. This structure was prepared by spin-casting and then drying at 100 °C on a hot plate for 30 min, with subsequent fuming reduction by hydrazine. As a result, the rGO gas sensor was fabricated without changes to the chemical functional groups on the rGO surfaces.

**Figure 2:**
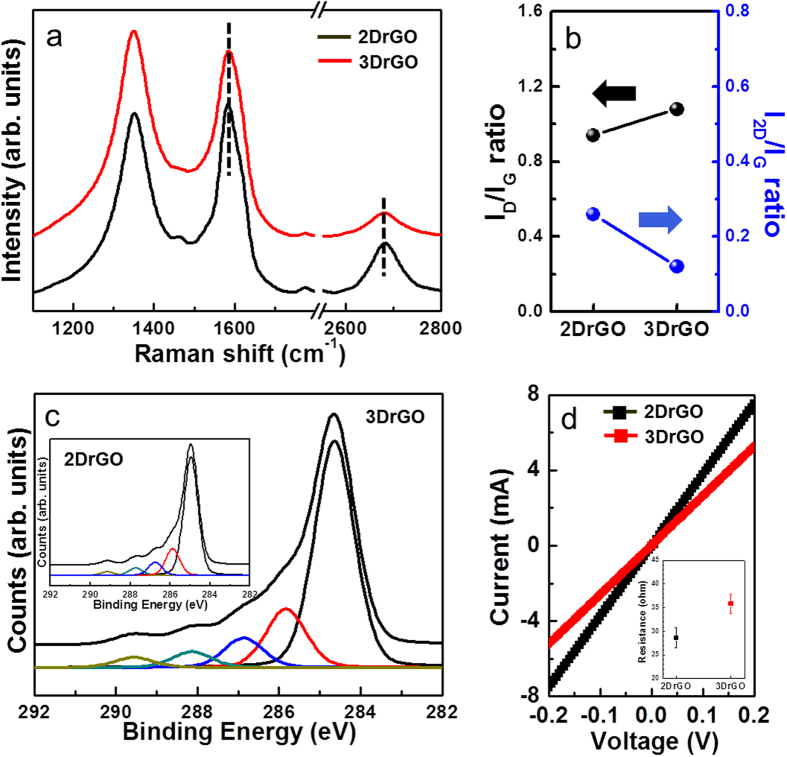
(**a**) Raman spectra of 2DrGO and 3DrGO (dashed line: G peak at 1592 cm^−2^ and 2D peaks at 2680 cm^−2^). (**b**) Intensity ratios for 2DrGO and 3DrGO (black arrow: I_D_/I_G_ ratio, blue arrow: I_2D_/I_G_ ratio). (**c**) XPS analysis of 3DrGO with 2DrGO in the inset. (**d**) I_ds_–V_ds_ characteristics for the interdigitated 2DrGO- and 3DrGO-based devices; inset: resistance of the samples.

**Figure 3:**
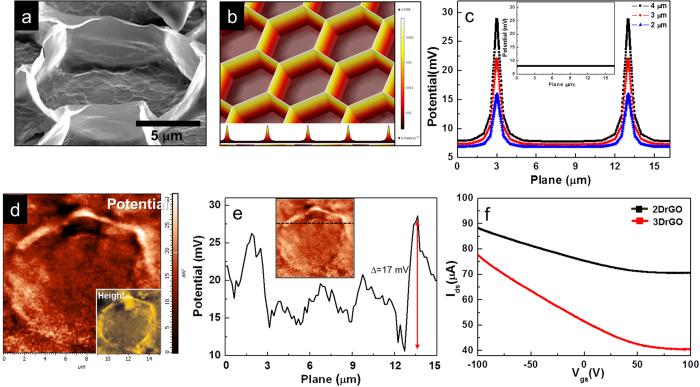
(**a**) FE-SEM image of hexagonal 3DrGO (scale: 5 μm). (**b**) Computational simulation of electric potential on a hexagonal structure at a specific height; inset: cross-sectional view of the electric potential. (**c**) Potential profiles of 3DrGO as a function of edge heights (2, 3, and 4 μm); inset: potential of the flat 2DrGO surface. (**d**) Measured charge distribution by SKFM on 3DrGO corresponding to Fig. 3a; inset: morphological image by AFM of the 3DrGO. (**e**) Measured potential profile for 3DrGO on the dashed line in the potential image of the inset. (**f**) I_ds_–V_gs_ characteristics for the 2DrGO and 3DrGO FET devices.

Structural changes, such as mechanical deformation, wrinkles, doping, and the number of layers of rGO, can be described by Raman spectra, as shown in Fig. 2a. Second-order zone boundary phonon (2D) peaks were observed at 2681 cm^−1^, with lower intensity ratio of I_2D_/I_G_ than single layered graphene, confirming heterogeneous structure^22^. In addition, the increased I_D_/I_G_ ratio of 3DrGO reveals a large number of structural changes or defects in the 3DrGO. However, the I_2D_/I_G_ of 3Dr_G_O is lower than that of 2DrGO in Fig. 2b. This may be due to the introduction of i) doping by chemical functional groups and ii) structural changes from the mechanical deformation of graphene. In general, the intensity of the 2D peak decreases with increasing doping ratio, which also shifts the G and 2D peak positions^23^. However, these peaks were not observed to shift in either sample, which rules out doping effects. Interestingly, the 2D peak position does not change between 2DrGO and 3DrGO, though the 3DrGO exhibited a larger full width at half-maximum (fwhm)—about 93 compared with a value of 68 for 2DrGO. This broadening of the 2D band arises from strain and defects, as reported previously^24^. For instance, a wrinkled graphene structure induces local, intra-layer strain, which causes a decreased relative intensity and broadening of the 2D peak^25^. In this case, the 3DrGO with corrugated surfaces of periodic architecture can be described in terms of a structural modification with local strain. This result demonstrates that the differences in rGO behavior are due to structural changes, not chemical functional groups. Figure 2c provides X-ray photoelectron spectroscopy (XPS) spectra for the two samples. The functionality of rGO is represented by the 2DrGO and 3DrGO. The C1s peaks of rGO and GO consist of four typical components arising from C = C (sp^2^, ~284.6 eV), C–C/C–N (sp^3^, ~285.8 eV), C–O (hydroxyl and epoxy, ~287.6 eV), C = O (carbonyl, ~288.3 eV), and O–C = O (carboxyl, 289.1 eV) bonds. With subsequent reduction by fuming hydrazine, the presence of oxygen functional groups was dramatically reduced. These results suggest that the structural changes of rGO were not affected by the functionality of oxygen moieties. Figure 2d shows the I_ds_–V_ds_ of the interdigitated rGO electrodes for both 2DrGO and 3DrGO. The resistance of 3DrGO (37 Ω) is slightly higher than that of 2DrGO (29 Ω), which agrees with the Raman analysis of structural changes of rGO. Although the morphology is significantly different, the resistance is only slightly altered because of the similar quantities of oxygen functional groups as insulating sites and the presence of nearly flat rGO films on the bottom layer of 3DrGO, as shown in Fig. 3a.

There are several issues involved in producing high-performance molecular sensors. A large surface area with corrugated surfaces facilitates the adsorption of large amounts of gas molecules and accelerated charge accumulation, which can improve gas detection in terms of sensitivity and fast-response time. In particular, the charge transfer induces significant changes in resistance due to the relation between electrostatic potential and charge density which can be described by Coulomb interaction. Also, the corrugated surfaces induce an inhomogeneous charge distribution. For instance, electron charge density increases on the rippled area of graphene surface^26^. In Fig. 3, significant morphological changes—including structural modification of rGO—are shown to induce an anisotropic charge distribution with accumulation of electrons on the regularly corrugated wall of the rGO layers. Figure 3a shows the hexagonal morphology of 3DrGO in an area with a diameter of 10 μm and wall height of 4 μm. The periodic, cratered morphology is formed by condensation and evaporation of water on the GO solution. The bottom layer of the structure is also formed by corrugated rGO, with a narrow diameter of 1 μm. The edges of the bottom layers are a few hundred nanometers tall, which is similar to the morphology of 2DrGO shown in Fig. 1c. In order to estimate the anisotropic charge distribution on the edge sites of 3DrGO, a computer simulation was carried out using COMSOL Multiphysics, which is based on Poisson’s equation (Fig. 3b)^27^. In other words, we generated a model using 3D simulation software based on the finite element method. In addition, the relative permittivity of the graphene was given as 2.5 in these calculations. The permittivity is based on the graphite. The value can be altered by morphology, reduction rate, and thickness of graphene layer etc. In particular, the potential ratio is crucial factor which depends on height of 3DrGO. Although the permittivity is different, the ratio is not altered. The electric potential distributions on the edge sites of 3DrGO are revealed to be higher than those along the bottom surface, which corresponds to a high density of states in the former. An electric potential of 0.1 V was applied to a virtual surface 3 μm above the graphene surface. This virtual surface, not shown in simulation images, has exactly same shape as the graphene surfaces. Figure 3c shows the potential profiles as a function of the height of the edge areas (for 2, 3, and 4 μm), where the electric potential is seen to increase with increasing edge height. The potential of the 3DrGO with a 4 μm edge height is five times greater than that of the 2DrGO, as shown in the inset of Fig. 3c. Notably, the corrugated 2DrGO film along the walls below the height of 3 μm is not affected by the anisotropic charge distribution. In other words, the ordered, protruding rGO surfaces increased the field enhancement factor compared to flat rGO films because of a high aspect ratio caused by geometrical modification^28^. In addition, the turn-on and threshold fields of the modified rGO surfaces were higher than those of flat rGO films. In Fig. 3d, the charge distribution of 3DrGO is confirmed by a surface-potential mapping using SKFM. This potential map resembles the morphology of 3DrGO, which was validated by atomic force microscopy (AFM), as shown in the inset of Fig. 3d. In addition, the wall thickness is about few tens nm in terms of staking of graphene layers during breath figure process. These SKFM and AFM maps were acquired simultaneously, with each pixel under precise control through a feedback-loop system. The Au-coated tips used had a 35 nm curvature radius and an approximate resonance frequency of 300 kHz. The measured potential is about 17 mV from the bottom to top position of the 3DrGO facet in Fig. 3e, which agrees with the simulation in Fig. 3c. In terms of localized electrons on corrugated graphene walls with symmetric morphologies, the carrier transport properties were altered compared with flat graphene surfaces. For instance, mesh-patterned graphene fabricated by ion beam etching has been shown to introduce differences in carrier-transport phenomena^29^. To demonstrate our samples’ electrical characteristics, an rGO field-effect transistor (FET) was fabricated on a 300 nm-thick SiO_2_ substrate with respective Cr and Au source and drain electrodes of 30 mm length and width. Figure 3f reveals the I_ds_–V_gs_ characteristics for both samples. It is clear that the strain and mechanical defects lower the on/off ratio due to the delocalization of electrons. In addition, the threshold voltage is similar for both samples. However, the on current of 2DrGO is higher than that of the 3DrGO, while the on/off ratio is lower. This is caused by mechanical deformation of graphene surfaces such as corrugation, breakage, and wrinkles^29^,^30^. This analysis confirms that the Fermi level of both samples is similar, which means an equal density of states, while the distribution of electrons on the graphene is different. Thus, the electrons on the 2DrGO surface are more uniformly distributed on than on the 3DrGO, which validates the electric-potential profile from the SKFM measurement. Therefore, our results confirm the anisotropic charge distribution of self-corrugated graphene, with a high density of states on the walls of 3DrGO.

**Figure 4:**
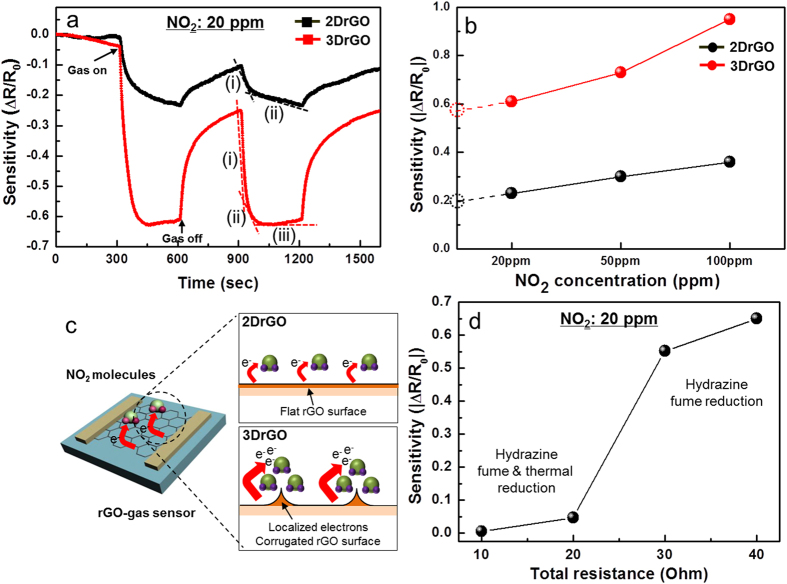
(**a**) Real-time response curve for the 2DrGO and the 3DrGO-gas sensors at a NO_2_ concentration of 20 ppm. (**b**) Sensitivity as a function of NO_2_ concentration. (**c**) Schematic diagram of an rGO-based gas sensor and its charge-transfer mechanism with NO_2_ gas molecules—2DrGO: uniformly distributed gas molecules on a flat rGO surface; 3DrGO: accumulated gas molecules on the localized electrons on the self-corrugated edges on the rGO surface, which shows a cross-sectional view of 3DrGO as in the inset of Fig. 3b. (**d**) Sensitivity change as a function of initial resistance on 3DrGO at 20 ppm gas concentration.

Figure 4a illustrates the gas-sensing performance of 2DrGO and 3DrGO at a NO_2_ gas concentration of 20 ppm. The gas sensitivity (S) is defined as S = ΔR/R_0_, where R_0_ is the initial resistance when exposed to pure N_2_ gas, and ΔR is the resistance change after exposure to the N_2_ and NO_2_ mixture. After exposure to NO_2_ gas at _2_0 ppm for 300 s, the resistance decreases due to the p-type property of rGO and the electron acceptor of the NO_2_ molecules^31^. The relative degree of sensitivity is defined as S = ΔR/R_0_ × 100 (%). The sensitivity of 3DrGO (65%) is roughly three times greater than that of 2DrGO (20%). Figure 4b shows the sensitivity as a function of NO_2_ gas concentration for 20, 50, and 100 ppm; the sensitivity increases linearly with increasing gas concentration. An extrapolation to lower gas concentrations predicts a sensitivity of about 0.2 at 1 ppm. This value is three times higher than that predicted for 2DrGO. The observed sensitivity values indicate that resistance increases with NO_2_ adsorption. Since the NO_2_ molecule is electrophilic, charges are expected to transfer from rGO to the physisorbed NO_2_, which confirms the p-type characteristic of rGO shown in Fig. 4c. The accumulated electrons on the corrugated surfaces would seem to cause an increased charge transfer compared with flat surfaces. This result demonstrates that such an anisotropic charge distribution—with electron accumulation on the rGO surfaces—can increase the probability of significant changes in electrical conductivity, which directly affects molecular sensitivity. In particular, 3DrGO exhibits a reversible fast response, unlike 2DrGO. This response mechanism comes from the charge transfer between gas molecules and active materials. For instance, metal-oxide semiconductors have a fast response at high temperature, which means that the activation energy of charge carriers increases with increasing temperature^32^. In this case, the activation energy of 3DrGO facilitates the fast adsorption of molecules due to charge accumulation in the corrugated walls of graphene shown in Fig. 3d.

The response curves for NO_2_ gas exposure can be separated into three regions, namely, (i) a sharp initial in gas response (fast-response region), (ii) a nearly linear intermediate region (slow-response region), and (iii) a region in which the sensor completely saturates (saturation region), as shown in Fig. 4a. In addition, the base-line before gas exposure is not stable. The drift effect might be occured by different amout of active sites with residual functional groups with resepct to both samples due to large surface area of 3DrGO compared to the 2DrGO. However, followed by NO_2_ gas exposure, the first and second cycle are stable. The base-line is saturated as shown in Fig 4a. The noise level is 0.001 which can be negligible in this case. The fast response comes from low-energy binding between sp^2^ carbon and gas molecules, while the slow response is due to mechanical defects or oxygen functional groups, as described earlier^33^. In this case, the slow response of 2DrGO is not well-saturated because of its strong interaction with high-energy binding sites when compared to the physisorption on the sp^2^ sites. Conversely, the 3DrGO exhibits a reversible response with the saturation region (iii). This indicates that a certain limit adsorption of gas molecules over the surface could be present. The excess NO_2_ molecules that did not reach the surface active sites of the sensor could then be expected to remain in the surrounding region. This establishes a constant gas response. These results demonstrate that a gas molecules interacting with high-energy binding sites during are adsorbed continuously during gas exposure. Meanwhile, the adsorption of gas molecules were saturated by accumulated gas molecules with fast response owing to high electric potential on the graphene.

Figure 4d shows that the initial resistance was significantly reduced for the fume-and-heat-treated rGO. However, the sensitivity of hydrazine-fume-reduced rGO is six times higher than that of the fume-and-heat-treated rGO, indicating that the initial resistance is dominated by the contact resistance between the Au electrode and the rGO. The fume-and-heat-treated rGO has lower initial resistance due to high reduction rate compared to the fume-treated rGO. The small amount of residual oxygen functional groups decrease bandgap of graphene, and then the metallic rGO decreases the contact resistance with metal electrodes. The dark current on resistive gas sensor can be generated in terms of electrical current on intrinsic devices aginst charge transfer from gas molecules to active layer. In particular, low contact resistance induces dark current. This strongly suggests that the presence of a sizable amount of initial resistance suppresses the dark current so that even a small amount of gas adsorption causes a large current increase. It is essential to maintain an optimal initial resistance to achieve high sensitivity in a molecular sensor^34^. In terms of structural modification of rGO surfaces, the exploitation of the anisotropic charge distribution of a 3DrGO-based gas sensor is a promising approach to achieving high-performance with a fast and stable response for practical use.

## Conclusions

We introduce self-corrugated, chemically modified graphene as a 3D structure that governs anisotropic charge distribution. This morphology can be formed by condensation and evaporation of water on a GO solution using the “breath figure” technique. In particular, the accumulated electrons on the edge sites of the corrugated graphene layers exhibit a strong electric field compared to flat graphene films. The sensitivity, stability, and fast response of 3DrGO were significantly improved in terms of gas-molecule detection. This method is a promising candidate among practical molecular sensors and flexible devices using chemically modified graphene without post-annealing process.

## Methods

### Preparation of 3DrGO on patterned electrodes

Graphenen Oxide (GO) was obtained by natural graphite (Alfa Aesar, 99.999% purity, –200mesh) according to a modified Hummers method^35^. Briefly, 20 g of graphite powder and 460 mL H_2_SO_4_ are mixed in a flask. Subsequently, 60 g of KMnO_4_ is slowly added for 1 h. For safety, stirring was carried out for 2 hours in ice bath. Again, the mixture is stirred vigorously for 24 hours at room temperature. DI (deionized) water is added and stirred for 10 min. A volume of 50 mL H_2_O_2_ (30 wt% aqueous solution) was then added, and the mixture was stirred for 2 h at room temperature. The mixture was precipitated and filtered to obtain the graphite oxide powder. This was then exfoliated into GO nanosheets in deionized water by homogenizer for 1 h^36^. The exfoliated GO solution was centrifuged at 10,000 rpm for 1 h to obtain almost single layered graphene. After decanting the supernatant, the sample was freeze-dried for a formation of GO powder. The GO was then dispersed in DI water with concentration of 1.5 g/l by conventional sonication for 10 min. Octadecylamine (ODA)-functionalized GO was dissolved in an organic solvent via ionic interactions. The ODA was dissolved in methyl ethyl ketone by bath sonication for 10 min, followed by added 20 vol % of ethanol. GO was functionalized with ODA via mixing a 2 mM ODA solution with the above GO dispersion. Subsequent stirring for 10 min, the ODA-functionalized GO nanosheets become aggregated. The addition of toluene in a volume amount equal to that of the GO dispersion resulted in the migration of the GO aggregates into the toluene phase. After stirring for 10 min, the solution is well dispersed in toluene with GO. The supernatant ODA-functionalized GO was then decanted delicately. Self-corrugated GO structures were achieved by ‘breath figure’ technique which comes from condensation and evaporation of solvent in terms of different vapor pressure between water and toluene. Specifically, 500 mL of an ODA-functionalized GO solution was drop-casted onto the patterned electrodes on SiO_2_ substrate with thickness of 300 nm. The interdigitated electrode was fabricated by simple shadow mask with deposition of metal source such as Cr and Au. The channel width and length of metal electrodes were 50 μm. The Cr/Au electrodes were deposited using thermal evaporator at 2 × 10^−6^ torr with a thickness about 20 nm and 100 nm respectively. In order to produce the regular structure of 3DrGO, humid air which has a relative humidity of 80% and a flow rate of 0.1 L/min was supplied from a nozzle affixed to a funnel. The vertical distance between the substrate and the end of the funnel was kept at 6 mm inside a desiccator. After complete evaporation of the solvents, vertically aligned patterns of the ODA-functionalized GO were obtained. Subsequently, the film was transferred to a furnace to eliminate the ODA from the GO. Pyrolysis was carried out at 250 °C for 1 h under atmospheric pressure. The self-corrugated rGO with uniform array structures were achieved after fume reduction using hydrazine monohydrate (N_2_H_4_) at 200 °C for 24 h. The 3DrGO gas sensor could be fabricated with high uniformity as shown in Fig. 1. In addition, there was no degradation of interdigitated electrodes during reduction and heat treatment.

### Characterization of the 3DrGO structure and its device performances

The morphologies of rGO were obtained by field emission scanning electron microscopy (FE-SEM, HITACHI S4800). The optical image of interdigitated electrodes for gas sensor was also revealed by optical microscope (Nikon Eclipse LV100). The structural and chemical characteristics in the 2DrGO and 3DrGO sheets were investigated by confocal Raman spectrometer (NTEGRA SPECTRA, NT-MDT) with an excitation wavelength of 532 nm and Rayleigh line injection filter with a spectral range of 100–3600 cm^−1^ for Stokes shift. To confirm the alteration in the atomic ratio of carbon to oxygen with presence of functional group with respect to the 2DrGO and 3DrGO samples after fuming reduction by hydrazine, the X-ray Photoelectron Spectroscopy (XPS: Thermo VG Scientific Inc. MultiLab2000) analyses were carried out on an X-Ray photoluminescence spectrometer with an Al cathode as the X-ray source with power of 150 W. The fitted peaks of XPS spectra were determined by considering a combination of Gaussian and Lorenzian distribution. The I_ds_-V_ds_ and I_ds_-V_gs_ characteristics were measured using a two-probe method (Keithley 4200-SCS). The total resistance with respect to 2DrGO and 3DrGO devices with interdigitated electrodes was measured by I_ds_-V_ds_. In order to measure the gating effect as depending of structures in Fig. 3f, the rGO field effect transistor (FET) was fabricated on a patterned electrode as described in the gas sensor with the same width and length. This is not interdigitated electrode but one pair electrode with source and drain. In order to confirm an anisotropic charge distribution on the rGO surfaces, the surface potential and morphology was measured by Scanning Kelvin Force Microscopy and Atomic Force Microscopy (SKFM, AFM: NTEGRA SPECTRA, NT-MDT). The system included simultaneously a tapping and mapping modes for measuring potential differences and morphology with 27 mm/s of scan speed. The potential measurement was carried out at room temperature with a humidity of 20%. In addition, the computational simulation which described in Fig. 3b,c was used by COMSOL Multiphysics which is the platform for physics-based modeling and simulation. The electric potential of 0.1 V was applied to a virtual surface in this case. The sensitivity and response time of interdigitated rGO gas sensors were obtained in a quartz chamber with nitrogen atmosphere. In addition, the NO_2_ gas was mixed with N_2_ gas. The concentration of NO_2_ gas with respect to 20, 50, 100 ppm was controlled by Keithley 2700 multimeter with solenoid valve system. The resistances of sensors were measured by Keithley 2000 multimeter, recording electrical resistance as a function of time for gas exposure. Two-probe DC resistance measurements was used by Au wires for current and voltage leads. The measured values were carried out under atmospheric pressure.

## Additional Information

**How to cite this article**: Yol Jeong, S. *et al.* Enhanced response and sensitivity of self-corrugated graphene sensors with anisotropic charge distribution. *Sci. Rep.* **5**, 11216; doi: 10.1038/srep11216 (2015).

## References

[b1] Geim A. K. & Novoselov K. S. The rise of graphene. Nat. Mater. 6, 183–191 (2007). 17330084 10.1038/nmat1849

[b2] Huang X., Qi X., Boey F. & Zhang H. Graphene-based composites. Chem. Soc. Rev. 41, 666–686 (2012). 21796314 10.1039/c1cs15078b

[b3] Schedin F. *et al.* Detection of individual gas molecules adsorbed on graphene. Nat. Mater. 6, 652–655 (2007). 17660825 10.1038/nmat1967

[b4] Robinson J. T., Perkins F. K., Snow E. S., Wei Z. Q. & Sheehan P. E. Reduced graphene oxide molecular sensors. Nano Lett. 8, 3137–3140 (2008). 18763832 10.1021/nl8013007

[b5] Booth T. J. *et al.* Macroscopic graphene membranes and their extraordinary stiffness. Nano Lett. 8, 2442–2446 (2008). 18593201 10.1021/nl801412y

[b6] Huang X. *et al.* Graphene-based materials: Synthesis, Characterization, Properties, and Applications. Small 7, 1876–1902 (2011). 21630440 10.1002/smll.201002009

[b7] Novoselov K. S. *et al.* Electric field effect in atomically thin carbon films. Science 306, 666–669 (2004). 15499015 10.1126/science.1102896

[b8] Berger C. *et al.* Electronic confinement and coherence in patterned epitaxial graphene. Science 312, 1191–1196 (2006). 16614173 10.1126/science.1125925

[b9] Kim K. S. *et al.* Large-scale pattern growth of graphene films for stretchable transparent electrodes. Nature 457, 706–710 (2009). 19145232 10.1038/nature07719

[b10] Stankovich S. *et al.* Graphene-based composite materials. Nature 442, 282–286 (2006). 16855586 10.1038/nature04969

[b11] Li D., Müller M. B., Gilje S., Kaner R. B. & Wallace G. G. Processable aqueous dispersions of graphene nanosheets. Nat. Nanotech. 3, 101–105 (2008). 10.1038/nnano.2007.451 18654470

[b12] Jeong S. Y. *et al.* Highly concentrated and conductive reduced graphene oxide nanosheets by monovalent cation-π interaction: Toward printed electronics. Adv. Funct. Mater. 22, 3307–3314 (2012).

[b13] Bagri A., Mattevi C., Acik M., Chabal Y. J. & Chhowalla M. Structural evolution during the reduction of chemically derived graphene oxide. Nat. Chem. 2, 581–587 (2010). 20571578 10.1038/nchem.686

[b14] Yavari F. *et al.* High sensitivity gas detection using a macroscopic three-dimensional graphene foam network. Sci. Rep. 1, 166 (2011). 22355681 10.1038/srep00166 PMC3240974

[b15] Han T. H., Huang Y.-K., Tan A. T. L., Dravid V. P. & Huang J. J. Steam etched porous graphene oxide network for chemical sensing. Am.Chem. Soc. 133, 15264–15267 (2011). 10.1021/ja205693t 21894991

[b16] Salehi-Khojin A. *et al.* Polycrystalline graphene ribbons as chemiresistors. Adv. Mater. 24, 53–57 (2012). 22113971 10.1002/adma.201102663

[b17] Deng S. *et al.* Reduced graphene oxide conjugated Cu_2_O nanowire mesocrystals for high-performance NO_2_ gas sensor. J. Am. Chem. Soc. 134, 4905–4517 (2012). 22332949 10.1021/ja211683m

[b18] Jeong H. Y. *et al.* Flexible room-temperature NO_2_ gas sensors based on carbon nanotubes/reduced graphene hybrid films. Appl. Phys. Lett. 96, 213105 (2010).

[b19] Lee S. H. *et al.* Three-dimensional self-assembly of graphene oxide platelets into mechanically flexible macroporous carbon films. Angew. Chem. Int. Ed. 49, 10084–10088 (2010). 10.1002/anie.201006240 21117056

[b20] Schonhorn H. *J.* Surface tension-viscosity relationship for liquids. Chem. Eng. Data 12, 524–525 (1967).

[b21] Dean J. A. in Lange’s Handbook of Chemistry 11th edn, Ch. 5, 1661 (McGraw-Hill, New York, 1967).

[b22] Torrisi F. *et al.* Inkjet-Printed Graphene Electronics. ACS Nano 6, 2992–3006 (2012). 22449258 10.1021/nn2044609

[b23] Das A. *et al.* Monitoring dopants by Raman scattering in an electrochemically top-gated graphene transistor. Nat. Nanotech. 3, 210–215 (2008). 10.1038/nnano.2008.67 18654505

[b24] Mohiuddin T. M. G. *et al.* Uniaxial strain in graphene by Raman spectroscopy: G peak splitting, Grüneisen parameters, and simple orientation. Phys. Rev. B 79, 205433 (2009).

[b25] Ismach A. *et al.* Direct chemical vapor deposition of graphene on dielectric surfaces. Nano Lett. 10, 1542–1548 (2010). 20361753 10.1021/nl9037714

[b26] Vazquez de Parga A. L. *et al.* Periodically rippled graphene: Growth and spatially resolved electronic structure. Phy. Rev. Lett. 100, 056807 (2008). 10.1103/PhysRevLett.100.056807 18352412

[b27] Reitz J. R., Milford F. J. & Christy R. W. in Foundations of Electromagnetic Theory 4th edn, Ch. 3, 57 (Addison-Wesley Publishing Co., Massachusetts, 1993).

[b28] Jeong H. J. *et al.* Self-organized graphene nanosheets with corrugated, ordered tip structure for high-performance flexible field emission. Small 9, 2182–2188 (2013). 23335443 10.1002/smll.201202143

[b29] Bai J., Zhong X., Jiang S., Huang Y. & Duan X. Graphene nanomesh. Nat. Nanotech. 5, 190–194 (2010). 10.1038/nnano.2010.8 PMC2901100 20154685

[b30] Hawker C. J. & Russell T. P. Block copolymer lithography: Merging bottom-up with top-down processes. MRS Bull. 30, 952–966 (2005).

[b31] Piloto C. *et al.* Highly NO_2_ sensitive caesium doped graphene oxide conductometric sensors. Beilstein J. Nanotechnol. 5, 1073–1081 (2014). 25161842 10.3762/bjnano.5.120 PMC4143126

[b32] Takao Y., Miyazaki K., Shimizu Y. & Egashira M. High ammonia sensitive semiconductor gas sensors with double-layer structure and interface electrodes. J. Electrochem. Soc. 141, 1028–1034 (1994).

[b33] Robinson J. T., Perkins F. K., Snow E. S., Wei Z. & Sheehan P. E. Reduced graphene oxide molecular sensors. Nano Lett. 8, 3137–3140 (2008). 18763832 10.1021/nl8013007

[b34] An K. H., Jeong S. Y., Hwang H. R. & Lee Y. H. Enhanced sensitivity of a gas sensor incorporating single-walled carbon nanotube-polypyrrole nanocomposites. Adv. Mater. 16, 1005–1009 (2004).

[b35] William J., Hummers R. E. & Offeman J. Preparation of gaphitic oxide J. Am. Chem. Soc. 80, 1339 (1958).

[b36] Jeong S. Y. *et al.* High-performance transparent conductive films using rheologically derived reduced graphene oxide. ACS Nano 5, 870–878 (2011). 21261292 10.1021/nn102017f

